# Clinical heterogeneity in chronic inflammatory demyelinating polyneuropathy: data-driven subtypes challenge current classification

**DOI:** 10.1007/s00415-026-14221-1

**Published:** 2026-10-22

**Authors:** Paula Quint, Felix Kohle, Menekse Oeztuerk, Jeremias Motte, Anna-Lena Fisse, Frauke Stascheit, Elisa Gilliam, Alice De Lorenzo, Ali Maisam Afzali, Katinka Fischer, Paul Disse, Christopher Nelke, Louisa Mueller-Miny, Anne K. Mausberg, Mark Stettner, Kalliopi Pitarokoili, Gerd Meyer zu Hoerste, Robert Brunkhorst, Marc Pawlitzki, Stefanie Schreiber, Achim Berthele, Bernhard Hemmer, Michael Schroeter, Sven G. Meuth, Christina B. Schroeter, Tobias Ruck

**Affiliations:** 1 Department of Neurology, Ruhr University Bochum, BG University Hospital Bergmannsheil, Bochum, Germany; 2 BG University Hospital Bergmannsheil, Heimer Institute for Muscle Research, Bürkle-de-La-Camp Platz 1, 44789 Bochum, Germany; 3 Department of Neurology, Faculty of Medicine, University of Cologne and University Hospital Cologne, Cologne, Germany; 4 Department of Neurology, Ruhr University Bochum, St. Josef-Hospital, Bochum, Germany; 5 Department of Neurology With Experimental Neurology, Charité - Universitätsmedizin Berlin, corporate member of Freie Universität Berlin and Humboldt Universität Zu Berlin, Berlin, Germany; 6 Neuroscience Clinical Research Center, Charité - Universitätsmedizin Berlin, corporate member of, Freie Universität Berlin and Humboldt Universität Zu Berlin, Berlin, Germany; 7 Department of Neurology, RWTH Aachen University Hospital, Aachen, Germany; 8 Department of Neurology, Institute for Experimental Neuroimmunology and, Technical University of Munich, School of Medicine and Health, Munich, Germany; 9 Department of Neurology, Medical Faculty and University Hospital, Heinrich Heine University Düsseldorf, Düsseldorf, Germany; 10 Department of Neurology, University Hospital Münster, Münster, Germany; 11 Department of Neurology, Center for Translational Neuro- and Behavioural Sciences (C-TNBS), University Hospital Essen, Essen, Germany; 12 Department of Neurology, Otto-Von-Guericke University Magdeburg, Magdeburg, Germany; 13 Department of Neurology, Technical University of Munich, School of Medicine and Health, Munich, Germany

**Keywords:** Chronic inflammatory demyelinating polyradiculoneuropathy (CIDP), Cluster analysis, Clinical phenotypes, Neuroimmunology

## Abstract

**Background:**

Chronic inflammatory demyelinating polyneuropathy (CIDP) presents with substantial phenotypic heterogeneity, yet current classifications do not adequately capture clinically meaningful subgroups. Thus, we aimed to discover novel, clinically meaningful CIDP subgroups using unbiased clustering.

**Methods:**

In this observational, multicenter retrospective cohort study we screened 425 patients diagnosed with typical CIDP or CIDP variants from 10 German centers. Standardized data including demographic, clinical, electrophysiological, laboratory, comorbidity, and treatment information were collected. 325 patients were then randomly assigned to the discovery cohort and 100 to the validation cohort. We performed unbiased clustering and post-hoc analysis to identify clusters and define subtypes.

**Findings:**

Four distinct clusters showed marked differences:

- *Cluster 1***:** Female patients with high symptom level, high disability scores, ANA positivity and autoimmune comorbidities
- *Cluster 2***:** Moderate to low symptom level with low disability scores, demyelination-dominant and less comorbidities
- *Cluster 3***:** High symptom level, axonal-demyelinating damage and cardiovascular comorbidities
- *Cluster 4***:** Multifocal CIDP with high disability scores as well as frequent ataxia and areflexia

Post hoc analyses showed marked differences in disease progression and therapeutic strategies, with clusters 1 and 4 showing particularly high rates of progression and disability.

**Interpretation:**

Unbiased clustering revealed four clinically meaningful CIDP subtypes, each exhibiting distinct presentations and trajectories. We identified two red flag subgroups suggesting that CIDP comprises biologically and clinically heterogeneous disease phenotypes. These findings may support more individualized monitoring and therapeutic strategies for high-risk patients and raise the possibility of distinct underlying pathomechanisms across CIDP subgroups.

**Supplementary Information:**

The online version contains supplementary material available at https://doi.org/10.1007/s00415-026-14221-1.

## Introduction

Chronic inflammatory demyelinating polyneuropathy (CIDP) is a chronic, immune-mediated demyelinating neuropathy of the peripheral nervous system. It is characterized by heterogeneous clinical presentations encompassing typical CIDP as well as several variants (distal, focal, multifocal, pure sensory, and pure motor forms) [1] These phenotypic differences, together with an incomplete understanding of pathophysiology, contribute to diagnostic challenges [2–4].

Current treatment strategies primarily include corticosteroids, intravenous immunoglobulins (IVIgs), and plasma exchange (PLEX) as first-line options [1, 5]. Beyond these treatments, novel therapeutic approaches for CIDP are emerging, including targeted strategies aimed at more individualized, mechanism-based disease control [3, 6]. However, treatment response is heterogenous with up to 50% of patients showing no objective response to initial therapy [7–10]. Although the 2021 European Academy of Neurology/Peripheral Nerve Society (EAN/PNS) guidelines provide updated diagnostic criteria, the conventional classification system does not reliably predict disease severity, course, or treatment responsiveness, [11] underscoring the need for refined approaches. Prior work has suggested that data-driven phenotyping may uncover clinically or electrophysiological meaningful subgroups [11, 12], yet existing studies were limited by small sample sizes, restricted variable sets or lack of external validation.

Given the substantial heterogeneity, we aimed to discover novel, clinically meaningful CIDP subgroups using unbiased clustering. Our objective was to integrate demographic, clinical, and diagnostic variables to identify subgroups with distinct characteristics and to explore whether these subgroups differ in disease course.

## Methods

### Patient recruitment

In this multicenter, retrospective analysis, we identified patients diagnosed with typical CIDP and CIDP variants according to the 2021 EAN/PNS criteria [1] at the University Hospitals in Aachen, Bochum (Bergmannsheil and St. Josef), Berlin, Cologne, Duesseldorf, Essen, Magdeburg, Munich and Muenster during the period of 2010 to 2024, excluding pure axonal forms. The study protocol was approved by the ethics committees of the Heinrich Heine University Duesseldorf (registration number 2022–1809) and was conducted in accordance with the committee’s guidelines and regulations and with the Declaration of Helsinki. Participating centers contributed data in accordance with applicable local ethical and regulatory requirements. Given the retrospective nature of the study and the use of pseudonymized data, individual informed consent and consent for publication were not obtained. Data collection and assessment followed the STROBE criteria.

### Data collection

We extracted standardized data including socio-demographics, symptoms, neurological examination, scores, comorbidities, laboratory and cerebrospinal fluid (CSF) profiles. Electrophysiological findings were categorized descriptively according to the predominant pattern on nerve conduction studies. A demyelinating pattern was defined by predominant features of impaired conduction, including reduced motor nerve conduction velocities, prolonged distal motor and/or F-wave latencies, conduction block, or temporal dispersion, where available. An axonal pattern was characterized predominantly by reduced compound muscle action potential (CMAP) and/or sensory nerve action potential (SNAP) amplitudes without predominant demyelinating features, while an axonal-demyelinating pattern was assigned when both features were present. This classification was used to describe the predominant electrophysiological phenotype and was not intended as a quantitative assessment of axonal loss. Needle Electromyography (EMG) findings were not systematically available and were therefore not included in the classification.

Testing for nodal and paranodal antibodies was available only in a small subset of patients (20% of all patients), with all available results being negative. Therefore, the antibody status was not included in further analyses. Moreover, detailed treatment histories were collected retrospectively according to routine clinical practice based on national guidelines and the individual treatment decisions of the treating physicians.

Data were collected using the respective hospital information system, excluding patients with > 50% missing data.

Clinical scores included the Inflammatory Neuropathy Cause and Treatment (INCAT) arm and leg disability score [13] and the Medical Research Council (MRC) sum score [14] at the time of first diagnosis and after 12, 24 and 36 months of follow-up, respectively.

Objective response to therapy was evaluated using the 2021 EAN/PNS criteria, [1] which include increases or decreases in clinical scores such as the INCAT or MRC sum score. Subjective response was defined based on the patient’s subjective assessment.

### Statistical analysis and clustering

Statistical analyses were performed in R version 4.4.3. The discovery cohort comprised all eligible patients (*n* = 325), excluding those assigned to the validation set (*n* = 100).

After harmonizing variables, we used hierarchical clustering with Gower distance to determine the optimal number of clusters. We then validated this structure using Partitioning Around Medoids (PAM) with *k* = 4 on the same distance matrix. Clustering was performed in a single run with a fixed random seed to ensure reproducibility.

### Cluster characterization

Clusters were described using mean ± standard error of the mean (SEM) for numeric data and counts and percentages for categorical variables. Between-cluster differences were tested with Chi-square or Fisher’s exact tests for categorical variables and Kruskal–Wallis tests for numeric/ordinal variables, with p values Benjamini–Hochberg adjusted (significance threshold: *p* < 0.05).

Cluster-defining variables were identified by enrichment relative to cluster centroids and visualized in a heatmap of cluster centroids, where variables with Benjamini–Hochberg false discovery rate (BH-FDR) < 0.05 were standardized to cohort-wide z-scores and averaged per cluster.

Discriminative performance of single variables was assessed by receiver operating characteristic (ROC) curve analyses using a one-versus-rest approach for each cluster. For each variable, the area under the curve (AUC) was calculated.

To test for potential center effects, the association between cluster membership and recruiting center was analyzed using chi-squared test of independence or Fisher’s exact test. Effect size was quantified using Cramér’s V.

### Validation in a separate cohort

A separate validation cohort of 100 patients was created by center-stratified random sampling, implemented as simple random sampling within each center using R’s pseudorandom number. For validation, we compared the variables used in the discovery cohort in a naïve PAM-derived classification within the validation set with an objective classification based on nearest-medoid assignment to the discovery solution using the Gower distance metric. Validity was quantified using Cohen’s kappa statistic and bootstrapped 95% confidence intervals.

### Post hoc analysis

Following cluster characterization, classification and regression trees (CART) were trained to identify clinical predictors of the previously defined PAM clusters. Missing data were handled using surrogate splits. The dataset was randomly split into training (80%) and test (20%) sets using stratified sampling. The final tree was optimized by 10-fold cross-validation and pruning based on cross-validation error. Model performance was assessed in the test set using confusion matrices, accuracy, sensitivity, and specificity.

Binary variables and INCAT scores not included in the clustering procedure were post hoc compared across clusters using chi-square or Fisher’s exact tests and Kruskal–Wallis tests, respectively. Pairwise comparisons using proportion tests or Wilcoxon tests as well as paired Wilcoxon tests for within-cluster longitudinal analyses were also used.

## Results

### Unsupervised clustering identified four distinct CIDP subgroups

Overall, data from 425 patients across ten centers were included, with 325 patients randomly assigned to the discovery cohort and 100 to the validation cohort. All patients fulfilled the 2021 EAN/PNS criteria for CIDP diagnosis.

**Fig. 1:**
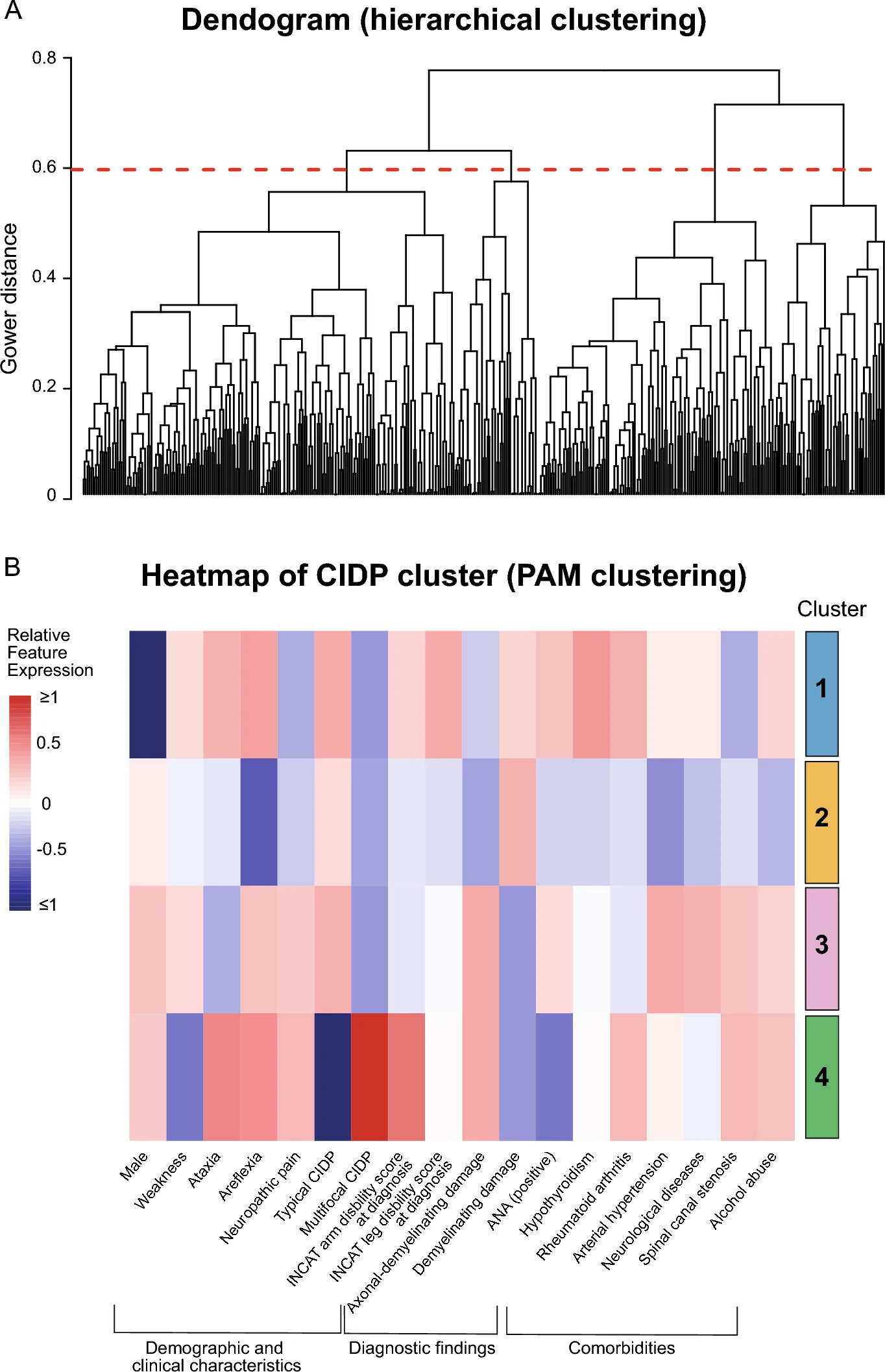
Dendrogram of Hierarchical Clustering and Heatmap of PAM clustering** A** Dendrogram of hierarchical clustering using the Gower distance (y- axis) across all individual patients in the discovery cohort (*n* = 325). The red line indicates a Gower distance threshold of 0.6. **B** Heatmap of five CIDP clusters obtained by PAM clustering. Each cell represents the cluster-wise mean z-score for variables that differed between clusters after Benjamini–Hochberg adjustment (FDR < 0.05). Rows correspond to clusters 1–4, and columns represent the included variables. The diverging color palette encodes deviation from the cohort mean (blue = lower, white = mean, red = higher). For binary variables, higher z-scores indicate higher prevalence; for continuous or ordinal variables, higher z-scores indicate higher average levels. ANA, antinuclear antibody; CIDP, chronic inflammatory demyelinating polyneuropathy; INCAT, Inflammatory Neuropathy Cause and Treatment; PAM, partitioning around medoids

In the discovery cohort, the hierarchical tree identified four distinct clusters that were used in the following PAM-clustering method (Fig. 1A). Cluster centroids differed particularly in the collected data including socio-demographics, symptoms, typical versus multifocal CIDP, clinical scores, diagnostic findings and comorbidities (Fig. 1B). Descriptive statistics for all collected variables are provided in Table 1.

**Table 1:** CIDP Clusters Identified by PAM Analysis

Variable	Cluster 1	Cluster 2	Cluster 3	Cluster 4
Total, No. (%)	51 (100)	120 (100)	107 (100)	47 (100)
Demographic and symptoms characteristics				
Male, No. (%)	13 (25)	93 (78)	94 (88)	40 (85)
Age at manifestation, mean (SEM), years	53 (2)	53 (1)	56 (1)	55 (1)
Age at diagnosis, mean (SEM), years	56 (2)	54 (1)	58 (1)	60 (1)
Family history of polyneuropathies, No. (%)	0 (0)	3 (4)	3 (6)	3 (7)
Sensory symptoms, No. (%)	51 (100)	115 (96)	105 (98)	45 (96)
Weakness, No. (%)	47 (92)	101 (84)	97 (92)	31 (67)
Fatigue, No. (%)	15 (33)	27 (27)	15 (15)	16 (35)
Ataxia, No. (%)	35 (70)	54 (46)	35 (33)	36 (78)
Areflexia, No. (%)	45 (90)	46 (39)	88 (84)	43 (93)
Tremor, No. (%)	6 (13)	19 (19)	14 (14)	16 (35)
Neuropathic pain, No. (%)	13 (27)	38 (32)	57 (56)	28 (61)
Disease course, No. (%)				
Typical CIDP	48 (94)	101 (84)	100 (93)	0 (0)
Multifocal CIDP	0 (0)	1 (1)	0 (0)	36 (77)
Pure sensory CIDP	2 (5)	9 (8)	3 (5)	5 (11)
Pure motor CIDP	0 (0)	3 (3)	1 (2)	0 (0)
Distal CIDP	0 (0)	2 (2)	4 (6)	5 (11)
Focal CIDP	0 (0)	4 (4)	0 (0)	0 (0)
Clinical scores at diagnosis, mean (SEM)				
MRC sum score	53 (1)	56 (1)	54 (1)	56 (1)
Vigorimetry (right hand)	48 (4)	72 (5)	66 (4)	73 (6)
Vigorimetry (left hand)	47 (4)	74 (5)	59 (4)	66 (5)
INCAT arm disability score	1.2 (0.2)	0.9 (0.1)	0.9 (0.1)	1.7 (0.3)
INCAT leg disability score	2 (0.2)	1.4 (0.1)	1.6 (0.1)	1.6 (0.3)
Diagnostic findings				
Axonal-demyelinating damage, No. (%)	29 (64)	61 (56)	91 (92)	25 (93)
Pure demyelinating damage, No. (%)	16 (36)	47 (43)	8 (8)	2 (7)
MGUS, No. (%)	6 (17)	9 (9)	5 (10)	3 (13)
Paraproteinemia, No. (%)	4 (11)	15 (18)	7 (8)	4 (15)
ANA (positive), No. (%)	12 (36)	12 (16)	26 (32)	0 (0)
ANCA (positive), No. (%)	3 (8)	2 (2)	2 (2)	0 (0)
Low vitamin B12, No. (%)	2 (6)	2 (3)	9 (11)	6 (24)
Low folic acid, No. (%)	6 (19)	1 (1)	6 (11)	2 (9)
Elevated liver enzymes, No. (%)	12 (30)	12 (11)	20 (32)	5 (21)
CK at diagnosis, mean (SEM), U/L	110 (15)	145 (10)	185 (28)	230 (65)
HbA1c at diagnosis, mean (SEM), %	5.8 (0.1)	5.8 (0.2)	6.1 (0.2)	5.7 (0.2)
CSF cell count at diagnosis, mean (SEM), cell/µl	2 (0.5)	11 (8)	3 (1)	3 (1)
CSF protein level at diagnosis, mean (SEM), mg/dl	72 (5)	101 (8)	79 (5)	65 (5)
Albuminocytological dissociation, No. (%)	33 (79)	78 (79)	75 (78)	19 (70)
Comorbidities, No. (%)				
Hypothyroidism	15 (31)	8 (7)	13 (13)	4 (14)
Rheumatoid arthritis	5 (11)	1 (1)	2 (2)	3 (11)
Arterial hypertension	25 (52)	29 (25)	71 (69)	12 (50)
Pulmonary diseases	6 (15)	14 (13)	12 (19)	1 (4)
Renal diseases	5 (12)	5 (5)	8 (13)	3 (12)
Neurological diseases ^1^	9 (22)	10 (9)	22 (35)	4 (17)
Restless legs syndrome	1 (2)	1 (1)	0 (0)	2 (8)
Depression	6 (15)	5 (5)	6 (10)	6 (25)
Malignancies	10 (21)	14 (12)	12 (12)	5 (18)
Diabetes mellitus type 2	10 (22)	15 (13)	22 (22)	4 (14)
Hyperlipidemia	13 (29)	12 (11)	24 (25)	7 (35)
Gastroenterological diseases	4 (10)	4 (4)	8 (13)	3 (12)
Spinal canal stenosis	0 (0)	7 (7)	13 (21)	5 (21)
Herniated disc	7 (18)	5 (5)	11 (17)	1 (4)
Nicotine abuse	9 (23)	18 (17)	29 (38)	6 (29)
Alcohol abuse	8 (20)	3 (3)	15 (21)	5 (23)
Treatment characteristics				
Time to first treatment, mean (SEM), months	4 (2)	5 (1)	7 (3)	12 (8)

Clinical data of each cluster. Results are shown as absolute numbers with their corresponding relative percentages of available data within each cluster or as mean  ±  SEM

*ANA* antinuclear antibody, *ANCA* antineutrophil cytoplasmic antibody, *CIDP* chronic inflammatory demyelinating polyneuropathy, *CK* creatine kinase, *CSF* cerebrospinal fluid, *HbA1c* hemoglobin A1c, *MGUS* monoclonal gammopathy of undetermined significance, *MRC* Medical Research Council, *PAM* partitioning around medoids, *SEM* standard error of the mean

^1^: Neurological diseases comprised any neurological comorbidity, including, for example, cerebrovascular, neurodegenerative, demyelinating, epileptic, headache, movement, and neuromuscular disorders, with the exception of polyneuropathies and restless legs syndrome

### Cluster Subgroups

#### Cluster 1 – Female patients with high symptom level, high disability scores, ANA positivity and autoimmune comorbidities

**Fig. 2:**
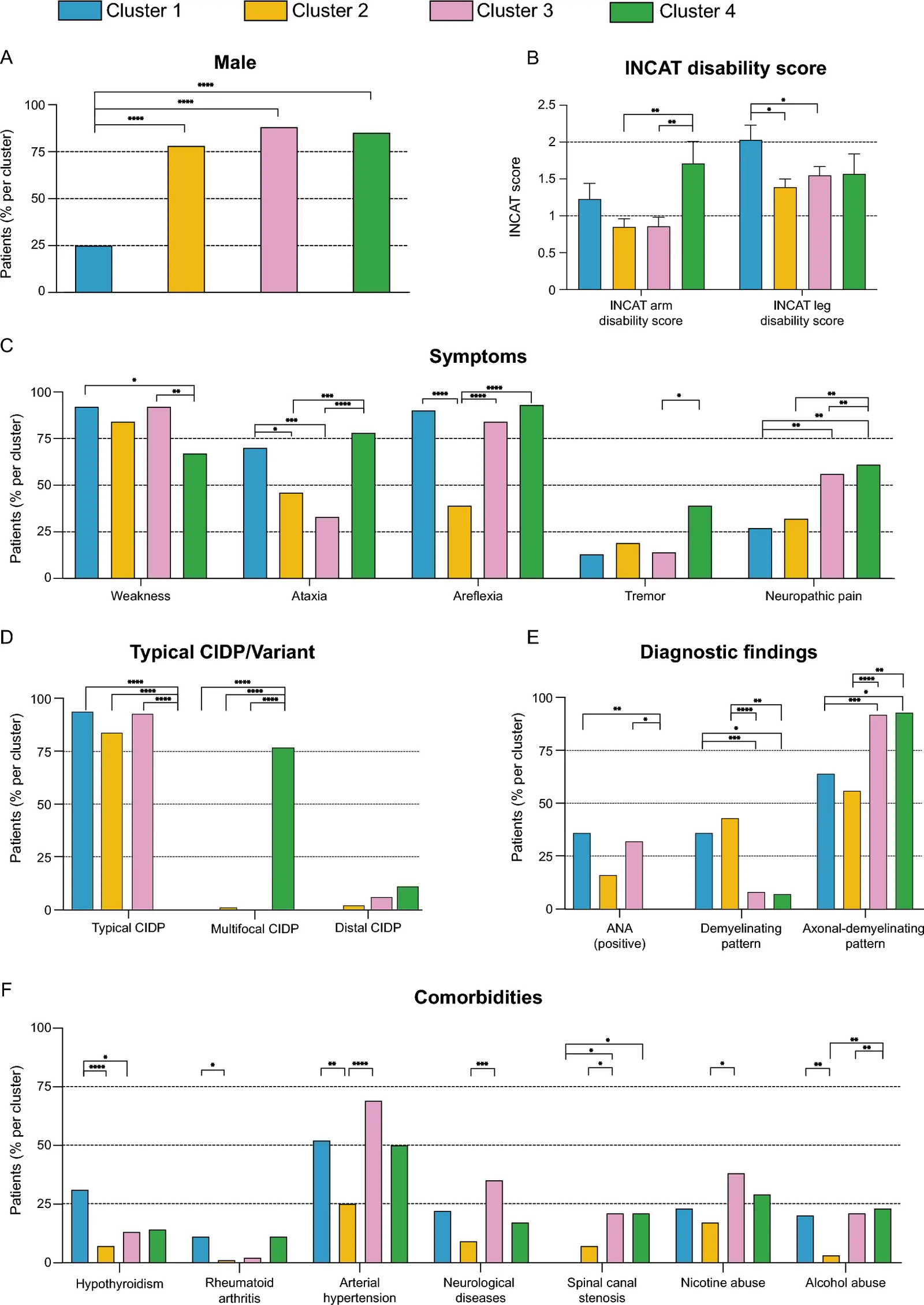
Clinical Characteristics of CIDP Clusters Male sex **A** INCAT disability score at diagnosis **B** symptoms **C** Typical CIDP/CIDP variant **D** diagnostic findings (**E**) and comorbidities (**F**) of each cluster displayed as relative percentage of all patients within the respective cluster. A p-value  ≥  0.05 was classified as not significant, *p*  <  0.05 (*), *p * <  0.01 (**), *p*  <  0.001 (***), and *p*  <  0.0001 (****) were considered significant. *ANA* antinuclear antibody, *CIDP* chronic inflammatory demyelinating polyneuropathy, *INCAT* Inflammatory Neuropathy Cause and Treatment

Cluster 1 included a total of 51 patients with the highest proportion of female patients (*n* = 38 (75%)) among all clusters. At diagnosis, INCAT leg disability remained higher than the other clusters, with a mean of 2 ± 0.2 points. While symptoms such as weakness (*n* = 47 (92%)), ataxia (*n* = 35 (70%)) and areflexia (*n* = 45 (90%)) were moderately to highly frequent, neuropathic pain was least frequent among the clusters (*n* = 13 (27%)), and patients were mainly classified typical CIDP (48 (94%) patients). In terms of diagnostics, patients in cluster 1 showed the highest level of ANA positivity (12 (36% of all tested) patients), with an axonal-demyelinating pattern mainly observed in neurography in 29 (64%) cases. Autoimmune comorbidities such as hypothyroidism (*n* = 15 (31%)) and rheumatoid arthritis (*n* = 5 (11%)) were most common among all clusters (Fig. 2).

**Fig. 3:**
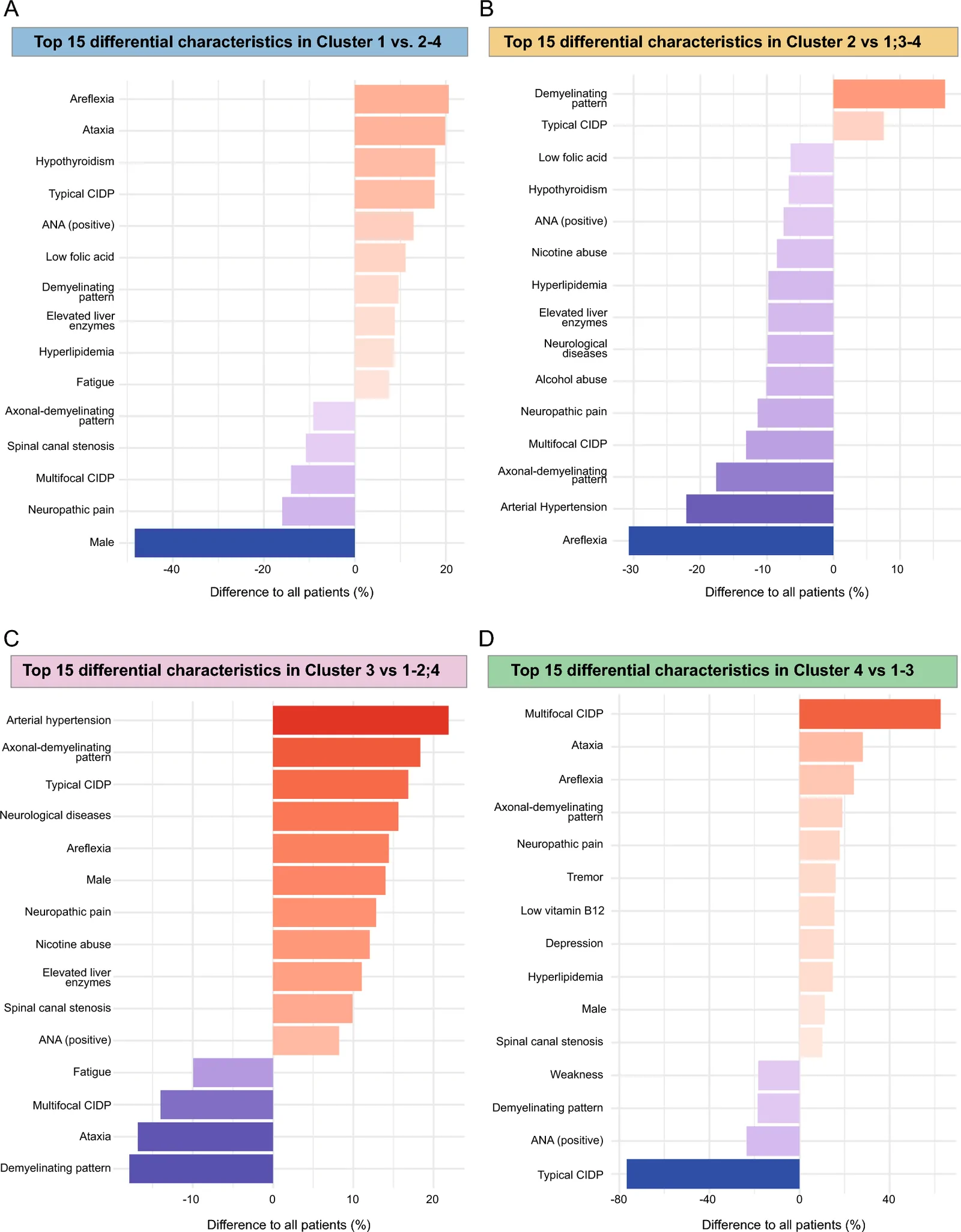
Top 15 Differential Characteristics of Each Cluster Top 15 differential characteristics between the cluster-specific prevalence of each cluster and the overall cohort prevalence. Only categorical variables are shown. Variables are plotted on the y-axis, and the difference relative to all patients (%) is plotted on the x-axis. *ANA* antinuclear antibody, *CIDP* chronic inflammatory demyelinating polyneuropathy

Through enrichment analysis, patients in cluster 1 were distinguished by their frequent areflexia, ataxia and hypothyroidism, while male patients as well as neuropathic pain and multifocal CIDP were uncommon characteristic features (Fig. 3A).

#### Cluster 2 – Moderate to low symptom level with low disability scores, demyelination-dominant and less comorbidities

Cluster 2, which represented the largest cluster, comprised 120 patients, including mainly male patients (*n* = 93 (78%)). At diagnosis, both INCAT arm and leg disability scores were lowest among all clusters (0.9 ± 0.1 and 1.4 ± 0.1). Weakness (*n* = 101 (84%)), ataxia (*n* = 54 (46%)) and neuropathic pain (*n* = 38 (32%)) showed moderate levels compared to the other clusters, while areflexia was least common in 46 (39%) patients. A typical CIDP was predominant (*n* = 101 (84%)), and neurography showed the highest proportion of pure demyelinating damage across the clusters in 47 (43%) cases. All captured comorbidities were significantly less frequent than in the other three clusters (Fig. 2).

Compared to the other clusters, differential characteristics included a high prevalence of demyelinating pattern and typical CIDP, while areflexia and arterial hypertension were uncommon (Fig. 3B).

#### Cluster 3 – High symptom level, axonal-demyelinating damage and cardiovascular comorbidities

107 patients were assigned to cluster 3, of which most patients were male (*n* = 94 (88%)). At diagnosis, INCAT arm and leg disability scores reflected moderate disability scores (0.9 ± 0.1 and 1.6 ± 0.1). Despites the lowest rates of ataxia in 35 (33%) patients, all other symptoms displayed moderate to high rates (weakness: 97 (92%) patients; areflexia: 88 (84%) patients; neuropathic pain: 57 (56%) patients), with the majority of patients diagnosed with typical CIDP (*n* = 100 (93%)). For diagnostics, positive ANA titers were common with 26 (32%) positive tests out of all tested, as well as an axonal- demyelinating pattern in neurography (91 (92%) patients). Comorbidities such as arterial hypertension (*n* = 71 (69%)), nicotine (*n* = 29 (38%) and alcohol abuse 15 (21%) as well as neurological diseases (*n* = 22 (35%)) were commonly observed (Fig. 2).

Differential characteristics included a higher prevalence of arterial hypertension, axonal-demyelinating damage and typical CIDP, whereas demyelinating damage, multifocal CIDP and ataxia were less common (Fig. 3C).

#### Cluster 4 – Multifocal CIDP and high disability scores together with frequent ataxia and areflexia

The fourth cluster comprised 47 patients and consisted predominantly of male patients (40 (85%) patients). With 1.7 ± 0.3 points, INCAT arm disability score reflected the highest arm disability at diagnosis. While weakness was least common in 31 (67%) patients, ataxia, areflexia and neuropathic pain were frequent in cluster 4 (*n* = 36 (78%), 43 (93) and 28 (61%)). Most patients showed a multifocal (*n* = 36 (77%)) variant, whereas no patient was diagnosed with typical CIDP. Neurography often showed an axonal-demyelinating damage in 25 (93%) cases, and none of the patients showed ANA positivity. Comorbidities, such as cardiovascular and autoimmune diseases, demonstrated moderate frequency (Fig. 2).

Compared to the other three clusters, differential characteristics included common multifocal CIDP and thus no typical CIDP, as well as frequent ataxia and areflexia while ANA positivity and an axonal-demyelinating pattern was less frequent (Fig. 3D).

### Prediction of cluster assignment through classification trees

**Fig. 4:**
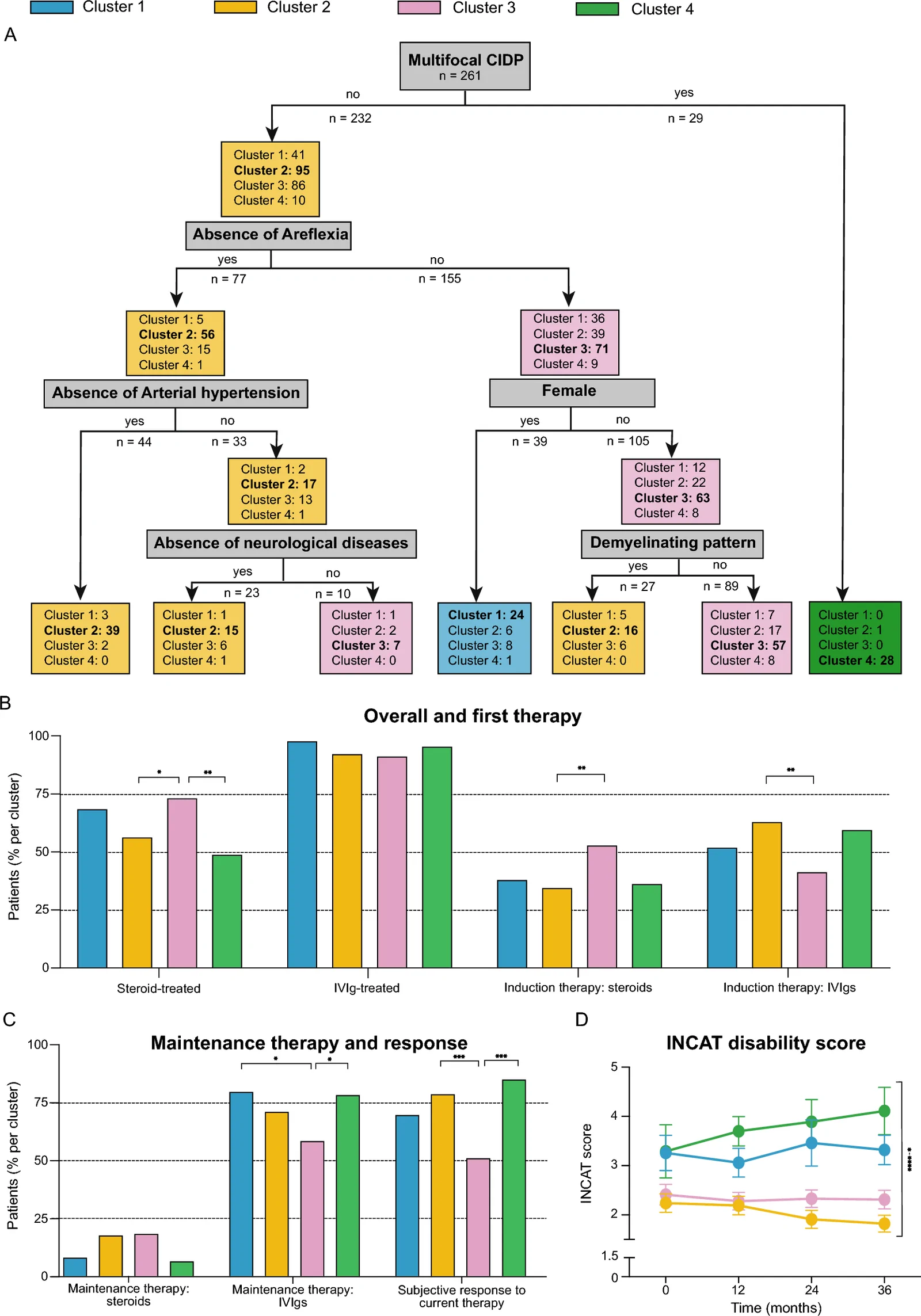
Classification Tree of Cluster Assignment and Post Hoc Analyses **A**: Decision trees illustrating the pathways leading to the four clusters. Each split represents a decision rule; terminal nodes display the sample size (n) for that path and the proportion of patients assigned to the respective clusters. 261 patients (80%) were randomly assigned to the training set used to construct the classification tree, while the remaining 20% (*n* = 64) were assigned to the independent test set for model evaluation. **B** **C:** Post hoc analyses of overall, first and maintenance therapy as well as therapy response. Data are presented as percentages for categorical variables. A p-value  ≥  0.05 was classified as not significant, *p*  <  0.05 (*), *p*  <  0.01 (**), *p*  <  0.001 (***), and *p * <  0.0001 (****) were considered significant. **D:** Comparison of total INCAT disability scores (arm + leg) between CIDP clusters over a follow-up period of 36 months. Significant differences in disability progression were observed between clusters at multiple time points. For reasons of clarity and readability, only selected results are displayed in the figure. Significant differences could be seen in INCAT total score at: diagnosis (1 vs 2 **; 1 vs 3 *; 2 vs 4*; 3 vs 4 *), 12 months (1 vs 2 **, 1 vs 3 *, 2 vs 4 *; 3 vs 4 *), 24 months (1 vs 2 ***; 1 vs 3 **; 2 vs 3 *, 2 vs 4 ***; 3 vs 4 **) and 36 months (1 vs 2 ***, 1 vs 3 **, 2 vs 3 *, 2 vs 4 ***, 3 vs 4 **). CIDP, chronic inflammatory demyelinating polyneuropathy; IVIgs, intravenous immunoglobulins; INCAT, Inflammatory Neuropathy Cause and Treatment

In Fig. 4A, a pruned classification tree including selected variables that showed significant differences in cluster analysis. 261 patients (80%) were randomly assigned to the training set used to construct the classification tree, while the remaining 20% (*n* = 64) were assigned to the independent test set for model evaluation. The tree achieved an overall accuracy of 81% in the test set, with an overall sensitivity of 84% and specificity of 93%.

The decision pathway associated with cluster 1 was characterized by the absence of multifocal CIDP, together with the presence of areflexia and female sex.

Cluster 2 comprised the largest number of patients and could be assigned through several distinct decision pathways. Main features included the absence of multifocal CIDP and areflexia, with either the absence of arterial hypertension or the presence of it together with the absence of neurological diseases.

Cluster 3 also displays the presence of multiple pathways, primarily characterized by the absence of multifocal CIDP, accompanied by areflexia, male sex and an axonal-demyelinating pattern.

Finally, multifocal CIDP emerged as the defining feature of cluster 4 and represented the sole decision criterion leading to this subgroup.

### Discriminative power

To quantify the discriminative power of the cluster-defining features, AUC values were calculated for each variable across the four clusters. Several clinical features showed a good discrimination (AUC ≥ 0.5), while an AUC ≥ 0.8 could be seen for typical and multifocal CIDP in cluster 4 (Supplemental Fig. 1).

### Validation and center effects

A validation cohort of 100 patients, including cases from all participating centers, was used for validation of the classification model. The validation demonstrated sufficient agreement between classifications, with an accuracy of 67% and a Cohen’s *κ* > 0.5 (95% CI 0.41–0.67).

A significant association between cluster assignment and recruiting center was observed (Chi-Square test, *p* < 0.05). The corresponding effect size, measured by Cramér’s V, indicated a moderate association between center affiliation and cluster membership (Cramér’s V = 0.48).

### Marked differences in therapy regimens

Post hoc, we analyzed differences in treatment strategies and responses among the four clusters. For overall treatment, significant differences could be seen for steroids, as steroids were less often used in cluster 2 (*n* = 62 (56%)) and 4 (*n* = 23 (49%)), whereas most patients in cluster 3 were treated with steroids (*n* = 77 (73.3%)).

Regarding first therapy, the preferred approach in cluster 3 was the use of steroids in 55 (53%) patients, whereas in the other clusters, it was less likely used (35% to 38%). Along with that, initiation therapy with IVIgs was less used in cluster 3 (*n* = 43 (41%)) while the other three clusters preferably used IVIgs as initiation therapy (52 to 63%).

For maintenance therapy, IVIgs were less likely used in cluster 3 (*n* = 61 (59%)) than in the other three clusters (71 to 80%). The objective treatment response to current therapy did not show significant differences, yet subjective therapy response was highest in cluster 4 (*n* = 35 (85%) patients) and lowest in cluster 3 (*n* = 44 (51.2%)).

In summary, overall steroid use as well as induction therapy were most common for cluster 3 in combination with lowest subjective treatment responses. Overall, maintenance therapy mainly included IVIgs with highest subjective responses for cluster 4 (Fig. 4b, c).

### Post hoc analyses of disease progression

Post hoc, we evaluated functional outcomes across clusters using longitudinal total INCAT scores, which were calculated from INCAT arm and leg scores. At diagnosis, both clusters 1 (3.3 ± 0.4) and 4 (3.3 ± 0.5) showed significantly higher scores, while cluster 2 had the lowest total scores (2.2 ± 0.2). Considering disease progression over a 36-month period as reflected by INCAT scores, cluster 2 showed favorable improvement. In contrast, clusters 1 and 3 remained largely stable, whereas cluster 4 demonstrated progressive deterioration over time (Fig. 4d).

### Summary of cluster characteristics

**Fig. 5:**
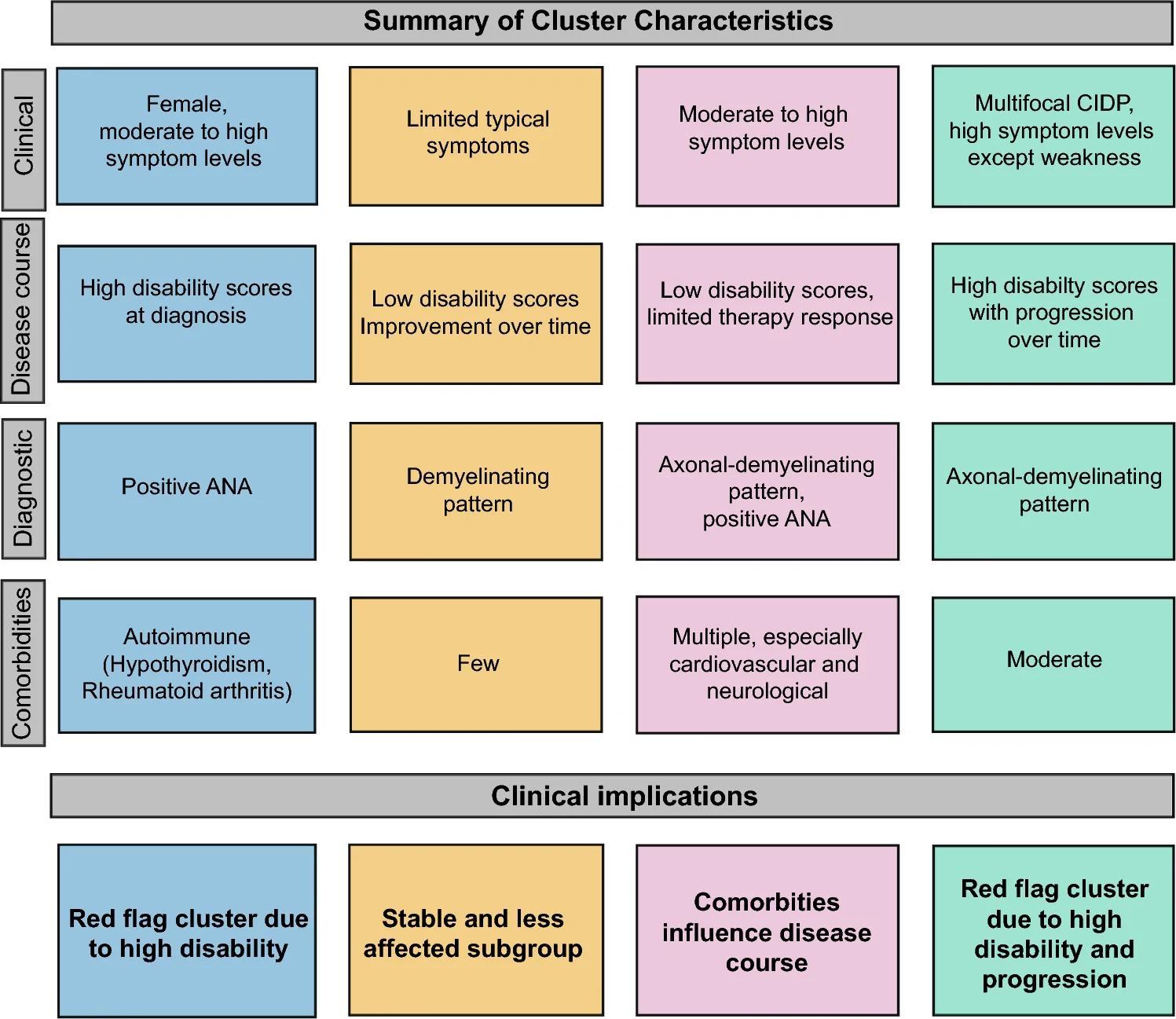
Summary of Cluster Characteristics and Clinical Implications Summary of the key clinical characteristics of the four clusters and their corresponding implications for clinical management. *ANA* antinuclear antibody, *CIDP* chronic inflammatory demyelinating polyneuropathy

In Fig. 5, we summarized main characteristic features of the four identified clusters through PAM and post hoc analyzes including clinical implications.

Cluster 1 included predominantly female patients on moderate to high symptom levels. At diagnosis, patients exhibited high disability scores, and positive ANA titers as well as autoimmune comorbidities were common. Due to high disability scores, patients were categorized as a red flag subgroup.

The largest cluster was identified as cluster 2, characterized by low levels of symptoms, low disability scores, and an improvement over time. In neurography, a demyelinating pattern was common and comorbidities were few. Consequently, this subgroup comprised stable and less affected patients.

For cluster 3, moderate to high symptom levels could be seen, with low disability scores at diagnosis but a limited subjective treatment response. Diagnostic features included a predominant axonal-demyelinating pattern in neurography and positive ANA titers. Comorbidities, especially cardiovascular comorbidities, were frequent, implicating a subgroup where comorbidities may influence disease course.

Finally, cluster 4 integrated multifocal CIDP patients with high symptom levels and high disability scores together with a progression over time. No ANA positivity could be seen, while neurography showed an axonal-demyelinating pattern and moderate comorbidities were associated. Thus, cluster 4 also reflects a red flag subgroup due to high disability and disease progression.

## Discussion

In this multicenter, data-driven analysis of 325 CIDP patients, we employed an unbiased clustering approach and identified four clinically meaningful subtypes with distinct clinical characteristics, validated in a separate cohort. Importantly, this multidimensional framework extends the current phenotype-based classification by identifying clinically relevant subgroups, including two distinct red flag clusters.

Regarding the phenotypes, cluster 1 was characterized by female patients with high symptoms levels, high disability scores, ANA positivity and autoimmune comorbidities.

Contrary to many other autoimmune disorders, CIDP is more prevalent in men than women [10, 15]. So far, sex-specific aspects of the disease have been insufficiently studied. Reported sex differences in several components of the immune system may contribute to the male predominance observed in CIDP, although the underlying mechanisms remain unclear [15]. Interestingly, the marked female predominance in cluster 1 together with high disability scores suggests that sex-specific disease characteristics may contribute to clinically relevant heterogeneity in CIDP and highlights the need for further studies investigating the role of sex in disease manifestation, progression, and treatment response.

Additionally, in cluster 1, over one third of tested patients showed ANA positivity. In the literature, positive ANA titers have been reported in a subset of CIDP patients [7, 16], yet with a lower prevalence than in cluster 1. Moreover, autoimmune comorbidities such as hypothyroidism and rheumatoid arthritis were more common in cluster 1, with a rheumatoid arthritis prevalence of 11%, markedly exceeding reported population estimates of 0.0–2.7% [17]. In general, coexisting immune disorders are common in CIDP [18, 19]. Even though several case studies of patients with either rheumatoid arthritis and CIDP or Hashimoto-Thyroiditis and CIDP exist [20, 21], systematic investigations of the prevalence and clinical relevance of these autoimmune comorbidities in CIDP subgroups are currently lacking.

Overall, cluster 1 represents a red-flag phenotype characterized by high disability, pronounced symptom severity, female predominance, autoimmune comorbidities, and ANA positivity, potentially reflecting additional autoimmune mechanisms and warranting careful differential diagnostic evaluation as well as further studies on biomarker-driven stratification and targeted therapies.

Cluster 2, which represented the largest cluster, features patients with moderate to low symptoms level, low disability scores, demyelination-dominant neurography and less comorbidities. Post hoc analyses of disease progression showed an improvement of INCAT scores over time, in contrast to the other three clusters. In the current literature, demyelinating features have been reported to be associated with high treatment response rates [12, 22], whereas early axonal loss was associated with disease severity and lack of treatment response [23, 24], corroborating our findings of cluster assignment. Thus, cluster 2 appears to represent a clinically stable subgroup without prominent red flag features, characterized by a comparatively uncomplicated and typical CIDP phenotype with lower overall disease burden and disability.

Moreover, cluster 3 comprised patients with high symptom levels, axonal-demyelinating damage and cardiovascular comorbidities. Overall, the presence of comorbidities in CIDP patients is common and often has an impact on therapy choices [18, 25]. However, data on cardiovascular comorbidities in CIDP are limited. An Italian cohort reported arterial hypertension in 35% of patients, comparable to the prevalence in the general population [18]. Moreover, available data on alcohol and nicotine abuse in CIDP are limited and do not suggest a significant impact on disease severity or progression [26, 27]. Given that corticosteroid use in the treatment of CIDP may cause or exacerbate hypertension [28], patients in cluster 3 reported highest overall and induction steroid use compared with the other clusters, accompanied by the lowest subjective response to current treatment. Still, it remains unclear whether arterial hypertension was steroid-induced, warranting further prospective investigation. Conclusively, arterial hypertension emerged as an important factor driving cluster separation and may contribute to disease trajectories in biologically susceptible subgroups supporting prospective evaluation of vascular-metabolic contributions to CIDP diagnosis and progression.

Finally, cluster 4 comprised primarily patients diagnosed with multifocal CIDP, with lowest rates of weakness and highest rates of other symptoms like ataxia, areflexia and neuropathic pain. Axonal-demyelinating damage was prevalent, and comorbidities were observed with moderate frequency. Post hoc analyses of disease progression showed highest disability scores, especially in the upper extremity, together with a progression over time, indicating that this subgroup may reflect a clinically distinct and potentially more dynamic disease phenotype, characterized as a red flag cluster. This observation is corroborated by the findings of previous studies, which indicate that multifocal CIDP may differ pathophysiologically from typical CIDP, with a possible predominant immune response of a cellular nature, accompanied by a disruption of the blood-nerve barrier at sites of conduction block [29, 30]. These findings may indicate that multifocal CIDP is not merely a distributional variant of CIDP, but may involve distinct immune-mediated mechanisms that contribute to ongoing disease activity and clinical worsening. Therefore, the co-occurrence of multifocality and progression in one cluster may point toward a subgroup requiring closer clinical monitoring and potentially earlier therapeutic adaptation.

In contrast to this enrichment of multifocal CIDP, the remaining clusters were predominantly composed of patients with typical CIDP, with smaller proportions of sensory, focal, distal, and motor variants that were less frequently represented in our cohort than reported in previous studies [11, 31, 32]. This difference may be related to cohort characteristics, retrospective phenotypic classification, or changes in phenotype over the disease course. However, the distribution of typical CIDP across three distinct clusters may further suggest heterogeneity within the typical phenotype.

Overall, these findings suggest that multidimensional clustering may provide a useful extension to the conventional classification of CIDP into typical and atypical phenotypes. Previous unsupervised clustering by Chang et al. [11] similarly identified distinct patient groups within CIDP, supporting heterogeneity beyond established phenotypic classifications. While their treatment-naïve cohort was clustered based on clinical, laboratory, and electrophysiological features, our multicenter real-world approach differed in clinical features including gender, symptoms, disease course, neurography, ANA positivity and comorbidities that were most important to distinguish CIDP patients. Despite differences in study population, included variables, and clustering methodology, both studies highlight the potential of data-driven approaches to capture clinically relevant dimensions of CIDP heterogeneity.

Importantly, our analysis identified two red-flag subgroups that require more personalized and closely monitored treatment (Fig. 5). These findings suggest distinct underlying pathomechanisms and corresponding clinical phenotypes, necessitating further prospective studies to validate and characterize these subgroups and potentially enable more individualized therapeutic approaches based on differing biological disease mechanisms. Moreover, the development of pragmatic assignment tools—such as neurophysiological, ultrasound or digital tools [33, 34]—will be essential to improve individualized monitoring and therapeutic decision-making in clinical practice.

This study has several limitations. Its retrospective design and methodological choices regarding feature selection, scaling, and mixed-data handling may have influenced cluster formation. In addition, although treatment variables were not included in the clustering procedure, prior treatment decisions may have influenced the clinical characteristics used for clustering and thereby indirectly affected cluster assignment. Furthermore, detailed information on treatment dosage and duration was not systematically available, limiting a more granular assessment of treatment effects.

A significant center effect suggests potential differences in referral patterns, diagnostics, and treatment practices between centers. However, the multicenter design enabled the inclusion of a large and clinically heterogeneous cohort, capturing diverse CIDP phenotypes. Although clustering patterns were reproduced in an independent validation cohort, the identified clusters should be considered exploratory and require external validation.

In conclusion, our data-driven framework identified four clinically interpretable CIDP subtypes with distinct disease trajectories, highlighting the need for more individualized therapeutic and monitoring strategies.

## Supplementary Information

Below is the link to the electronic supplementary material.

Supplementary file1 (PDF 451 KB)

## Data Availability

The data that support the findings of this study are available from the corresponding author upon reasonable request.
