# Supplementary material for: Clinical heterogeneity in chronic inflammatory demyelinating polyneuropathy: data-driven subtypes challenge current classification
Source: J Neurol. 2026 Oct 22;273(11):681. doi: 10.1007/s00415-026-14221-1 (PMC13690833; doi:10.1007/s00415-026-14221-1)
Supplement: Supplementary file 1 [file 415_2026_14221_MOESM1_ESM.pdf]

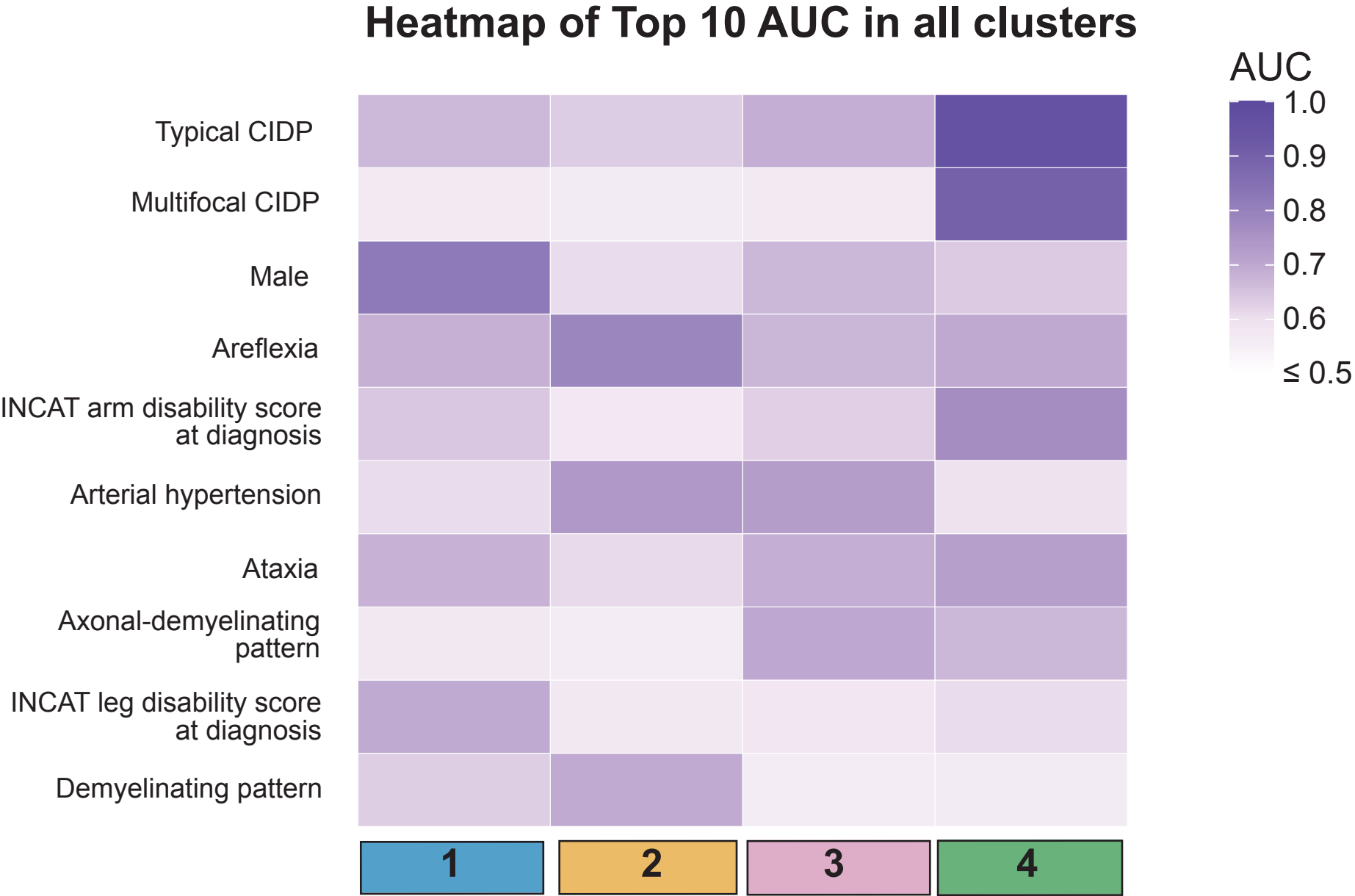
Heatmap of Top 10 AUC in all clusters
Typical CIDP
Multifocal CIDP
Male
Areflexia
INCAT arm disability score at diagnosis
Arterial hypertension
Ataxia
Axonal-demyelinating pattern
INCAT leg disability score at diagnosis
Demyelinating pattern
1
2
3
4
AUC
1.0
0.9
0.8
0.7
0.6
≤ 0.5
